# TRPV1 in pain control from the brain

**DOI:** 10.18632/oncotarget.13316

**Published:** 2016-11-11

**Authors:** Daniel Martins, Marta Silva, Isaura Tavares

**Keywords:** pain, development

Despite of the intense research confirming the funding Hippocratic idea that “*Men ought to know that from the brain, and from the brain only, arise our(…)pains*”, pain modulation from the brain remains a cutting-edge issue in neurobiology. It is now accepted that the brain has the capacity to modulate the magnitude of perceived pain, depending on individual and contextual factors (such as stress, anxiety or attention).

Pain modulation from the brain is under a continuous balance between inhibition and facilitation. Increased descending facilitation appears to account for chronic pain installation. The periaqueductal gray (PAG) is a gateway control center that conveys to the spinal cord the modulatory inputs triggered from cortical and subcortical areas. The modulatory influences exerted by the PAG are typically relayed by the rostroventromedial medulla (RVM), an area that arbors neurons associated with pain inhibition or pain facilitation. Although pain modulation from the brain may differ accordingly to pain modality, an important role for the RVM in pain modulation has been ascribed [1].

While better known for its role in pain transduction in the periphery, recent reports proposed that the transient receptor potential vanilloid type 1 (TRPV1) may also participate in pain regulation from the brain. Noteworthy, Silva et al., 2016 [2] provided new insights about the role of TRPV1 in pain modulation from the brain by showing, for the first time, that the receptor plays a role in RVM-mediated pain control during chronic pain (namely in an animal model of metabolic neuropathy - the streptozotocin diabetic rat). In this work, we demonstrate that mRNA levels of TRPV1 in the RVM of diabetic neuropathic animals increase about 30 times when compared to age-matched healthy control animals. This huge increase of TRPV1 in the RVM of diabetic rats seem to be accompanied by a doubling of the levels of fatty acide amide hydrolase (FAAH), an enzyme involved in the catabolism of several of the TRPV1 endogenous ligands. Although the activity of FAAH was not directly evaluated, this increase in FAAH expression is likely to have functional consequences, once endogenous TRPV1 ligands typically catabolized by FAAH decrease in the RVM of diabetic animals. The administration of a TRPV1 agonist in the RVM of diabetic neuropathic induced a decrease of nociceptive responses in diabetic rats but had no effect in healthy animals, which nicely matches the very low expression of TRPV1 detected in the RVM of these control animals. Altogether, these data support that the TRPV1 may play a role in descending modulation from the RVM during diabetic neuropathy by mechanisms that do not seem to be functionally relevant in heathy control conditions.

**Figure 1:**
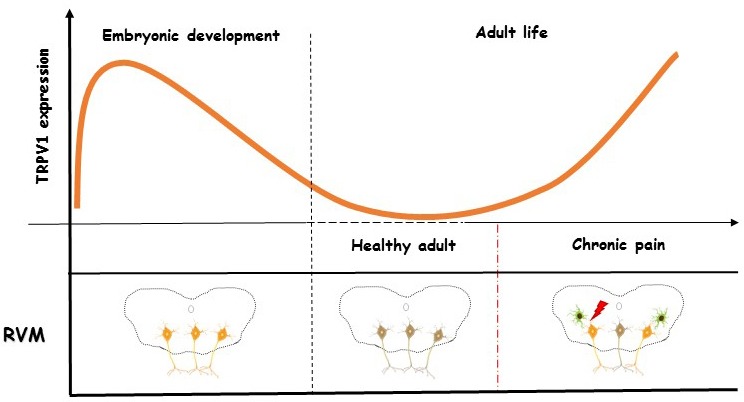
Temporal profile TRPV1 expression in the RVM; TRPV1 expression peaks during embryonic development, decaying to almost undetectable levels in adult life Chronic pain during diabetic neuropathy represents a stressor that forces RVM neurons to recapitulate the embryonic expression of TRPV-1, representing a mechanism of neural plasticity.

Chronic pain is known to induce a plethora of plastic changes in the brain as a mechanism of adaptation to the continuous arrival of nociceptive input. We propose that upregulation of TRPV1 in the RVM of diabetic rats may represent an example of those processes. Neurobiologists that study the role of TRPV1 in the brain [3], have been thrilled by situations in which the low or almost undetectable levels of this receptor in baseline conditions increase during “stressful” conditions, as it appears to occur with the excessive transmission of nociceptive information during chronic pain. In the other side, an elegant previous study genetically modifying the *TrpV1* locus to reveal its distribution and fate map suggested that expression of TRPV1 in some brain areas (namely the RVM) may be target of a developmental restriction. TRPV1 expression can, thus, exert a transient role in embryonic development of some brain areas, then decaying to undetectable levels postnatally [4]. Supporting this idea, recent studies showed that TRPV1 plays a key role during neuronal differentiation and neurogenesis. Although the precise mechanisms behind this interesting feature of TRPV1 remain to clarify, current research has been concentrating efforts in shedding light on the putative implications of TRPV1 in regeneration of injured neurons and its proadaptive network integration [5]. Inspired by these findings, we propose that chronic pain could represent a mechanism that challenges brain neurons to recapitulate some of the plastic features of embryonic development, such as TRPV1 signaling. Supporting this idea, a recent report showed that the developmental factor Prrxl1 can increase its expression in the dorsal root ganglia during inflammatory pain [6]. Prrxl1 participates in the establishment of the synaptic dorsal root ganglia -spinal cord circuits and its expression was described to decay to almost negligible levels after birth. Is this upregulation reflecting a similar phenomenon to that observed for TRPV1 in the RVM? Can the “embryo” takes hold the reins again? TRPV1 and Prrxl1 may, therefore, represent good proof-of-concepts (Figure 1).

Activation of the neuro-immune response has been assumed to induce the re-expression of extraembryonic-like axes within injured tissues, thus favouring their re-development [7]. Accumulating evidence suggests that neuroinflammation in the nervous system has an important role in chronic pain. We previously reported an increase of oxidative stress damage and microglia activation in the RVM of neuropathic diabetic animals [8]. Can this activation of immune responses account for reactivation of developmental programs (such as TRPV1) in brain areas during chronic pain, as a response to oxidative damage?

The evidences supporting activation of embryonic programs during chronic pain are still scarce and require further investigation. Nevertheless, we highlight the putative interest of developmental recapitulation as a new line of research to provide new insights on chronic pain mechanisms and potential drug targets.

Isaura Tavares: Department of Experimental Biology, Faculdade de Medicina, Institute of Molecular and Cell Biology, Institute of Investigation and Innovation in Health, Universidade do Porto, Portugal
